# Association of RAGE gene polymorphism with Type-2 diabetes mellitus in local population

**DOI:** 10.12669/pjms.341.14359

**Published:** 2018

**Authors:** Saba Zulfiqar, Fatma Hussain, Amer Jamil, Nisar Ahmed

**Affiliations:** 1 Saba Zulfiqar, Molecular Biochemistry Laboratory, University of Agriculture Faisalabad, Pakistan; 2 Fatma Hussain, Molecular Biochemistry Laboratory, University of Agriculture Faisalabad, Pakistan; 3 Amer Jamil, Molecular Biochemistry Laboratory, University of Agriculture Faisalabad, Pakistan; 4 Nisar Ahmed, Center of Agricultural Biochemistry and Biotechnology (CABB), University of Agriculture Faisalabad, Pakistan

**Keywords:** PCR, Polymorphism, RFLP, T2DM, Variants

## Abstract

**Objectives:**

Type-2 diabetes mellitus (T2DM) is an endocrine disease having a significant genetic component. Polymorphisms of many genes may affect hereditary vulnerability of the disease that is characterized by insulin resistance and islet disorder. As the genetic basis of T2DM can vary between ethnic groups, it is important to investigate the genetic link of T2DM in Pakistani populace. This study was aimed to assess the association of receptor for advanced glycation end product (RAGE) gene polymorphism (-429T>C) with Type-2 diabetes mellitus within local populace.

**Methods:**

Genomic DNA was isolated by following kit protocol. Genotyping of the RAGE gene was studied by PCR-RFLP on genomic DNA. All research work was done in molecular biochemistry laboratory (MBL), University of Agriculture Faisalabad and Postgraduate Laboratory, The University of Faisalabad, Pakistan from December 2016 to July 2017.

**Results:**

We found distribution of -429T>C genotypes between T2DM and healthy controls as 24.7% (tt), 24.7% (Tt) and 50.7% (TT). The outcomes were highly compatible statistically.

**Conclusion:**

The techniques of PCR and RFLP when performed simultaneously can be helpful in tracing vital information regarding polymorphism of AGE receptor.

## INTRODUCTION

Diabetes mellitus (DM) is a polygenic disorder. It may also be called a heterogeneous abnormality. It needs lifelong care. This problem is characterized by chronic symptoms such as high blood glucose level. The metabolism of macromolecules is defective due to impaired hypoglycemic hormone secretion, its action or both. Multifactorial etiology points towards the lifestyle as major predisposing factor.^1^

The cell types which express RAGE include endothelium, monocytes/macrophages, T-lymphocytes, neuronal cells and glomerular epithelial cells.^2^,^3^ Although RAGE genes has been topic of interest in many studies involving various ethnic populations to explore its link with Type-2 diabetes and post diabetic manifestations but results observed were conflicting.^4^,^5^

Genetics being an important player for the progression of this disease identifies individuals at risk of developing T2DM. Purpose of present study was to assess the two RAGE genetic polymorphisms found exclusively in promoter region and to reveal how RAGE genotypes and allelic distribution directly affects disease prevalence in Pakistani population.

## METHODS

### Sample collection

Blood samples from 50 normal individuals and 100 patients suffering from Type-2 diabetes mellitus (T2DM) admitted to Allied Hospital, Faisalabad and Madina Teaching Hospital Faisalabad, Pakistan were collected. All the molecular research was conducted in Clinico-Biochemistry Laboratory (CBL) and Molecular Biochemistry Laboratory (MBL), Department of Biochemistry, University of Agriculture Faisalabad (UAF), Pakistan from December 2016 to July 2017.

### DNA extraction

Genomic DNA was extracted by using blood genomic DNA isolation kit (FavorPrep Blood Genomic DNA Extraction Mini Kit, Biotech Corp, Taiwan). Two hundred μL samples (whole blood) were shifted to a centrifuge tube followed by the addition of 200μL FABG buffer and 20μL proteinase K sequentially. It was mixed thoroughly by pulse-vortexing. The mixture was incubated at 60°C for 15 minutes to lyse the contents of the sample. During this incubation, sample was vortexed after every 3-5 minutes. It was followed by addition of 200 μL of ethanol (96-100 %) and pulse-vortexing for 10 seconds.

The whole mixture with sediments was shifted to a FABG mini column placed in a tube and centrifuged for one minute at 6000 × g. The FABG, mini.column was placed in another tube and 40μL of W1 (buffer) was added. It was placed in a separator for 30 seconds at optimum speed (18,000 × g) and the flow-through was discarded. It was followed by the addition of 750μL of washing buffer to the FABG mini column. It was centrifuged for 30 seconds at full speed and eluent was discarded. Further centrifugation for three minutes at full speed was done in order to dry the column. This step inhibited subsequent enzymatic reaction. The FABG mini column was placed in an elution tube and 60 ~ 200 μL of elution buffer (pH 7.5- 9.0) was put on the membrane of FABG mini column. The FABG mini column was placed in a standing position for three minutes. For elution it is necessary to dispense elution solution onto the membrane center and it should be completely absorbed. Finally it was centrifuged at 6000 × g speed for one minute to elute total DNA. The extracted DNA was stored at 4°C.

### Quantification of DNA

The quantity of the DNA was determined by measuring the absorbance of DNA samples (A_260_/A_280_). Quality of the DNA was assessed on 0.8% agarose gel. By using 1X TAE buffer, the gel was run (80 V), stained in ethidium bromide for 15-20 minutes and documented in gel documentation system.

### Single nucleotide polymorphism (SNP) of RAGE gene

SNP was selected from promoter region of RAGE gene for analysis in diabetic patients as well as in healthy subjects.

### RAGE gene primer

The primer used for the selected variant (-429T/C (rs1800625) of RAGE gene is mentioned below.

F: GGGGCA GTTCTCTCCTCACT and

R: GGTTCAGGCCAGACTGTTGT

### Primer designing

Primers were designed using “primer 3” software (http://primer3.wi.mit.edu/).

### Polymerase Chain Reaction (PCR)

PCR conditions were optimized for annealing temperature and magnesium concentration. A PCR kit provided by Thermo SCIENTIFC™ (U.S.A) was used. All PCRs was done in duplicates to minimize the variations. The composition of a 25 μL PCR mixture was 2.5 μL (2 mM) dNTPs, 2.5 μL 10X *Taq* DNA polymerase buffer, 2.5 µL (25 mM) MgCl_2_, 1 µM forward primer, 1 µM reverse primer, 0.5 μL*Taq* DNA polymerase, 1.0 μg genomic DNA and nuclease free water to make up the volume. PCR reaction was run in a thermal cycler with following conditions: 95°C for 5 minutes followed by 25 cycles of 94°C for 30 seconds, 53°C for 30 seconds, 65°C for 4 minute with a final extension of 5 minute at 65°C.

### Enzymatic digestion

After amplification with PCR, all amplified fragments were subjected to digestion using specific enzyme under optimum conditions of temperature and time allowing access to genotype of each individual. The selected restriction enzyme was: *Alu*I (rs1800625) for RAGE polymorphic sites -429T/C.^5^

## RESULTS

### -429T>C polymorphism in control group and T2DM patients

Genetics being an important player for the progression of this disease identifies individuals at risk of developing T2DM. Purpose of present study was to assess the RAGE genetic polymorphisms found exclusively in promoter region and to reveal how RAGE genotypes and allelic distribution directly affects disease prevalence in Pakistani population.

**Fig. 1:**
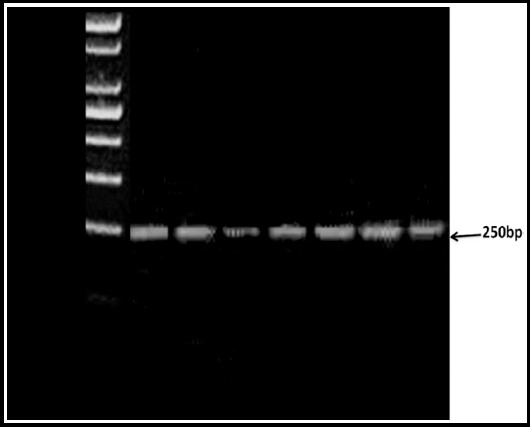
Electrophoresis of 2.5% agarose gel with PCR product loaded. -429 RAGE gene polymorphisms, M: negative control, 1, 2, 4, 5, 6, 7 fragments with 250 bp.

**Fig. 2:**
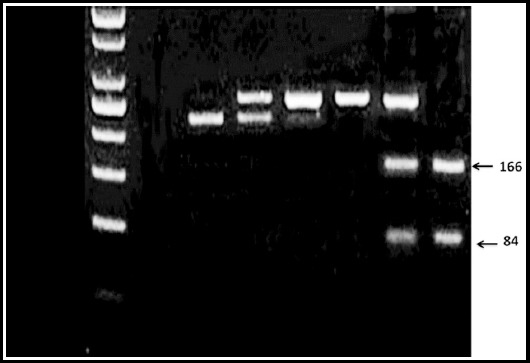
Electrophoresis of 2.5% agarose gel with AluI enzymatic digestion of RAGE gene polymorphisms. Lane 1: negative control, Lane 2: homozygous TT genotype (250 bp), Lane 3 heterozygous Tt genotype (166 and 84 bp), Lane 4: homozygous TT genotype (250 bp), Lane 5: homozygous TT genotype (250 bp), Lane 6: heterozygous Tt genotype (166 and 84 bp), Lane 7: Tt genotype (166 and 84 bp).

The digested products were directly electrophoresed on 2.5% (w/v) agarose gel with ethidium bromide and the results were recorded using a gel documentation system. Enzymatic digestion was done for verification of all the distinctive genotypes.

Fragments of 166 bp and 84 bp for -429T>C allele were produced after digestion with *Alu*I. The size of unrestricted fragment was 250bp.

The -429T>C genotype fragments indicated heterozygosity (Tt) after digestion with restriction enzyme *Alu*I (rs1800625). -429T>C genotypeis present in the promoter region of RAGE gene; hence polymorphism of this region may have an impact on RAGE protein structure and may be involved in pathogenesis of T2DM. However, Kang *et al*. (2012) demonstrated that this variant of RAGE gene had no impact in T2DM pathogenesis and metabolic parameters to justify the hypothesis that -429T>C may be prone to cause diabetic micro-vascular complications.^6^ In view of current study -429T>C allelic frequencies were statistically non-significant between control and T2DM groups. Distribution of -429T>C genotypes between T2DM and healthy controls were 24.7% (tt), 24.7% (Tt) and 50.7% (TT).

## DISCUSSION

The RAGE gene (chromosome 6; 6p21.3) is located near a histocompatibility complex.^7^ Promoter RAGE polymorphisms -429T>C (rs1800625) and 63 bp deletion (-345 to -407 bp) are associated with an increase in RAGE expression^8^ and diseases related with immune system such as systemic lupus erythematosus, Crohn's disease^9^ and DM.^10^

Engelen et al.^11^ depicted that the metabolic impairment due to hyperglycemia influence RAGE polymorphism. As T2DM is a multidimensional disease, a polymorphism as prominent etiological factor in one ethnic group may not have the same link in other ethnic populations.^12^ Furthermore, Li et al.^13^ stated that the incidence of RAGE gene polymorphism may vary depending upon type of diabetes and ethnic background. Receptor for AGEs is mostly expressed at the level of transcription of mRNA and translation of protein during initial developmental phases in lungs under normal physiological circumstances. Tissues of heart, kidney and brain show maximum expression of RAGE. Very low degree of RAGE expression is observed in macrophages and monocytes in normal physiological state. Tissue degeneration happens because of persistently accumulating AGEs.

Impact associated with RAGE gene polymorphism on various biochemical and metabolic parameters was elaborated to assess their link to T2DM problems (Table-I). Non-significant association was observed between -429T>C site and T2DM complication groups. Comparable results were presented by Ng et al.^5^ as they found no association of -429T>C with T2DM in Malaysian population. Many genetic and epigenetic factors may be linked with T2DM and post diabetic complications, but pathophysiology remains unclear.^14^

**Table-I:** Genotype and allelic frequencies of -429T>C in diabetic and controls.

Genotype		Subject		Total
		Diabetic	Normal	
tt	N	23	14	37
	%	23.0%	28.0%	24.7%
Tt	N	26	11	37
	%	26.0%	22.0%	24.7%
TT	N	51	25	76
	%	51.0%	50.0%	50.7%
Total	N	100	50	150
	%	100.0%	100.0%	100.0%

Data expressed as X^2^: 0.561^NS^; p-value: 0.756, NS: non-significant.

Current project traced the relationship of RAGE gene polymorphism with for pathogenesis of T2DM. Present study did not demonstrate any association of -429T>C variant of RAGE gene with biochemical parameters as well as with diabetic complications among Pakistani diabetic population.

### Authors' Contribution

**FH** conceived the study.

**SZ** did data collection and manuscript writing along with statistical analysis & editing of manuscript.

**AJ** provided the guidelines for practical genomic work.

**NA** facilitated in sorting out the technical issues regarding this project.

**SZ** takes the responsibility and is accountable for all aspects of the work in ensuring that questions related to the accuracy or integrity of any part of the work are appropriately investigated and resolved.

## References

[1] Leslie RD, Palmer J, Schloot NC, Lernmark A (2016). Diabetes at the crossroads: relevance of disease classification to pathophysiology and treatment. Diabetologia.

[2] Wendt TM, Tanji N, Guo J, Kislinger TR, Qu W, Lu Y (2003). RAGE drives the development of glomerulosclerosis and implicates podocyte activation in the pathogenesis of diabetic nephropathy. Am J Pathol.

[3] Rong LL, Yan SF, Wendt T, Hans D, Pachydaki S, Bucciarelli LG (2004). RAGE modulates peripheral nerve regeneration via recruitment of both inflammatory and axonal outgrowth pathways. FASEB J.

[4] Santos KG, Canani LH, Gross JL, Tschiedel B, Souto KEP, Roisenberg I (2005). The -374A allele of the receptor for advanced glycation end products gene is associated with a decreased risk of ischemic heart disease in African-Brazilians with type 2 diabetes. Mol Genet Metab.

[5] Ng ZX, Kuppusamy UR, Tajunisah I, Fong KCS, Chua KH (2012). Association analysis of S429T/C and S374T/A polymorphisms of receptor of advanced glycation end products (RAGE) gene in Malaysian with type 2 diabetic retinopathy. Diabetes Res Clin Pract.

[6] Kang P, Tian C, Jia C (2012). Association of RAGE gene polymorphisms with type 2 diabetes mellitus, diabetic retinopathy and diabetic nephropathy. Gene.

[7] Gonzalez I, Romero J, Rodriguez BL, Perez-Castro R, Rojas A (2013). The immunobiology of the receptor of advanced glycation end-products: trends and challenges. Immunobiology.

[8] Hudson BI, Stickland MH, Futers TS, Grant PJ (2001). Effects of novel polymorphisms in the RAGE gene on transcriptional regulation and their association with diabetic retinopathy. Diabetes.

[9] Wang Z, Hu J, Fan R, Zhou J, Zhong J (2014). RAGE gene three polymorphisms with Crohn's disease susceptibility in Chinese Han population. World J Gastroenterol.

[10] Picheth G, Costantini CO, Pedrosa FO, Martinez TLR, de Souza EM (2007). The -374A allele of the receptor for advanced glycation end products (RAGE) gene promoter is a protective factor against cardiovascular lesions in type 2 diabetes mellitus patients. Clin Chem Lab Med.

[11] Engelen L, Ferreira I, Gaens KH, Henry RM, Dekker JM, Nijpels G (2010). The association between the -374T/A polymorphism of the receptor for advanced glycation end products gene and blood pressure and arterial stiffness is modified by glucose metabolism status: the Hoorn and CODAM studies. J Hypertens.

[12] Niu W, Qi Y, Wu Z, Liu Y, Zhu D, Jin W (2012). A meta-analysis of receptor for advanced glycation end products gene: four well-evaluated polymorphisms with diabetes mellitus. Mol Cell Endocrinol.

[13] Li Y, Yang C, Ma G, Gu X, Chen M, Chen Y (2014). Association of polymorphisms of the receptor for advanced glycation end products gene with COPD in the Chinese population. DNA Cell Biol.

[14] Lindholm E, Bakhtadze E, Sjogren M, Cilio CM, Agardh E, Groop L (2006). The -374 T/A polymorphism in the gene encoding RAGE is associated with diabetic nephropathy and retinopathy in type 1 diabetic patients. Diabetologia.

